# Medical Reform

**Published:** 1842-04

**Authors:** 


### Medical Reform.

The following is an extract from an excellent Memorial recently addressed to Sir James Graham, by the Council of the North of England Medical Association:

"The grievances of medical practitioners may be briefly summed up, as consisting—in the unfair competition arising from the dissimilarity in the qualifications of candidates for medical practice and honours—the general neglect of their interests, ensuing from the want of a proper organization in the professional body throughout the empire—the absence of a protective power for the qualified practitioner, against the encroachments of unqualified and ignorant pretenders to medical knowledge—the exclusion of their *members* from all control over the management of most of the medical corporations.

"The powers vested in the corporations are suited to the accomplishment of certain objects *only*. They are of a limited character, and not adapted to the superintendence of the profession *as a body*, how well soever they might, *with certain modifications*, promote the welfare of the particular departments to which they belong.

"For the *general* direction and control of medical affairs in each division of the United Kingdom, your memorialists are of opinion that a presiding body or council is required, which shall be responsible to the Crown and to the profession.

"To obviate the disadvantages arising from the dissimilitude in the regulations of the various examining and licensing boards, and to ensure the general competency of all future candidates for medical practice, your memorialists conceive that a definite qualification should be established, without which no person should receive a license to practice; that such qualification should be made uniform throughout England, Scotland, and Ireland; and that such license should convey the right to practice every branch of the profession, and in any part of the United Kingdom.

"The possession of a national license to practice would by no means interfere with the existing classes of physicians, surgeons, and general practitioners, although a contrary statement has been pertinaciously adhered to by the opponents of medical reform, and by those who are interested in the continuance of the present state of affairs; neither would it take from any university or college the privilege of educating students, or of granting degrees, diplomas, or other honorary distinctions. The national license would certify, that the licentiate had been educated and examined in all the branches of medical science, to what branch soever he might more especially devote his attention, either in study or in practice; and although the London corporations collectively have declared that 'a course of study and a test of competency adapted to each particular branch of the profession, affords a much surer guarantee for a high standard of qualification in each branch, than could be obtained by a course of study and examination common to all,' the most eminent members of their councils have individually pronounced, that the education of the physician and surgeon should be the same; and as the general practitioner combines in his practice, the practice both of the physician and the surgeon, it follows that all practitioners should in the first instance, be similarly qualified. Degrees and titles in medicine and in surgery (with admission into the Colleges of Physicians and Surgeons) would, under such an arrangement, be (as they now are) open to those who might be anxious to procure them. The honorary diplomas granted by the College of

Surgeons in London have increased in number since the passing of the Apothecaries' Act, although the course of study and examinations requisite for their attainment are entirely self-imposed on the part of candidates. Physicians residing in the provinces have also, at various times, connected themselves with the London College of Physicians, although the authority of that college is virtually restricted to the metropolis and its immediate neighbourhood."

---

### BOOKS RECEIVED FOR REVIEW.

